# Improved pregnancy rate with administration of hCG after intrauterine insemination: a pilot study

**DOI:** 10.1186/1477-7827-8-18

**Published:** 2010-02-23

**Authors:** Ilkka Y Järvelä, Juha S Tapanainen, Hannu Martikainen

**Affiliations:** 1 Department of Obstetrics and Gynaecology, Oulu University Hospital, PO Box 5000, FIN-90014 Oulu, Finland

## Abstract

**Background:**

In natural cycles, women conceive when intercourse takes place during a six-day period ending on the day of ovulation. The current practice in intrauterine insemination (IUI) cycles is to perform the IUI 24-36 hours after the hCG administration, when the ovulation is already imminent. In this study hCG was administered after the IUI, which more closely resembles the fertilisation process in natural cycles.

**Methods:**

All the IUIs performed since the beginning of 2007 were analysed retrospectively. Our standard protocol has been to perform the IUI 24-32 hours after hCG administration. From the end of 2008, we started to inject hCG after the IUI at random. The main outcome measure was the result of a urinary pregnancy test. Generalized Estimating Equations (GEE) was used to identify independent factors affecting the cycle outcome.

**Results:**

The analysis included 228 cycles with hCG administered before and 104 cycles hCG administered after the IUI. The pregnancy rates were 10.9% and 19.6% (P = 0.040), respectively. Independent factors (OR, 95% CI) affecting the cycle outcome were sperm count (2.65, 1.20-5.81), number of follicles > 16 mm at IUI (2.01, 1.07-3.81) and the time of hCG administration (2.21, 1.16-4.19).

**Conclusion:**

Improved pregnancy rate was observed with administration of hCG after IUI.

## Background

Intrauterine insemination (IUI) is a common treatment. The pregnancy rate in IUI cycles with partners' sperm has varied between 11.4% and 12.6% during 2001-2004 in Europe [1] and the multiple birth rate between 11.2% and 13.1%. As reported by the ESHRE Capri Workshop Group on IUI [1], despite the use of clomiphene/gonadotrophins to induce multiovulation and the preparation of the semen sample, the pregnancy rates in IUI cycles are not significantly better than the results achieved after ordinary or timed intercourse. In fact, IUI has not been classified as an assisted reproductive technique (ART) despite its wide use [2].

The ESHRE Capri Workshop Group wanted to clarify the role of individual topics in the effectiveness of IUI treatment. One of the topics was the timing of the insemination. Accordingly, in the majority of the studies included in the analysis, the insemination was performed 32-36 hours after hCG administration [1]. However, it appears that among healthy women, the best chance to become pregnant is if intercourse occurs up to six days before ovulation [3]. If this is applied to the IUI protocol, the hCG should be injected after the insemination rather than before it. In this study we wanted to evaluate the effect of postponing the hCG injection until after IUI.

## Methods

### Subjects

We analysed the data on IUI cycles carried out between January 2007 and September 2009 at the Department of Obstetrics and Gynaecology, Oulu University Hospital, Oulu, Finland. In all of these cycles, a clomiphene citrate/FSH/hCG stimulation protocol and a standard IUI technique with partner's sperm were used.

The study couples had at least 1 year of infertility and had undergone a basic infertility evaluation consisting of anamnesis, semen analysis using WHO guidelines and hysterosonosalpingography. Prolactin and TSH concentrations were assessed if the menstrual period was irregular and polycystic ovarian syndrome was not observed. The upper age limit for the treatment was 40 years. IUI was the first treatment offered and it was performed up to two times in all the couples without severe oligospermia or bilateral tubal patency. If this proved unsuccessful, we continued with controlled ovarian hyperstimulation and IVF/ICSI.

### Ovarian stimulation

All women in the study underwent ovarian stimulation using clomiphene citrate and FSH. They were given 50-100 mg of clomiphene citrate on cycle days 3 to 7, followed by 75-150 IU FSH daily. Ovarian and endometrial responses were monitored by vaginal ultrasonography on cycle days 9 to 12. IUI was then scheduled at the point when it was estimated that the largest follicles would have reached 17-18 mm in diameter. If more than three follicles > 16 mm existed at the time of IUI, the excessive follicles were either emptied or the cycle was abandoned. The abandoned cycles were not included in the analysis. IUI was performed even when the follicles had already been ruptured by the time of IUI.

### Sperm preparation and intrauterine insemination

Semen samples were collected by masturbation after 2-4 days of sexual abstinence. After liquefaction and initial sperm analysis, the standard gradient centrifugation technique was used for preparation, employing SpermGrad™ gradient material in G-IVF™ Plus-medium (Vitrolife Ab, Gothenburg, Sweden).

Intrauterine insemination was performed using an intrauterine catheter with a 1-ml syringe. The catheter was passed through the cervical canal and the sperm suspension expelled into the uterine cavity. Insemination volume was 0.5 ml. The women remained supine for 5-10 min after IUI.

### Timing of hCG injection

The standard protocol in our unit has been to inject hCG (5000 IU) when at least one follicle has reached 17 mm in mean diameter. The hCG was injected in the morning and the IUI was performed next day, 24-32 hours after the administration of hCG. We have not requested patients to wake up during night-time for the injection, since there seems to be no difference in the pregnancy rate in IUls performed at 24 hours versus 36 hours after hCG [4]. All patients in 2007 and the majority of those in 2008 were treated according to this protocol. From the end of 2008, we started to give the hCG injection 3-5 minutes after IUI procedure. The IUI procedure was performed when the largest follicle had reached 17 mm in diameter. No randomisation in the timing of hCG injection was performed at any time in this pilot study. The vast majority of patients treated in 2009 had the hCG injection after the IUI. No luteal support was given to any of the patients. If menstruation was delayed after IUI, a urinary pregnancy test was performed. If the test was positive, a transvaginal ultrasonographic examination was performed at pregnancy week 7. Clinical pregnancy was confirmed if intrauterine gestational sac with heart beat was detected.

### Statistical analysis

Differences between groups were tested using Student's t test and the χ^2 ^test. We used Generalized Estimating Equations (GEE) to identify independent factors contributing to the success (positive pregnancy test and clinical pregnancy rate) of the IUI cycle. This model takes into account that there may more than one observation per patient. The factors selected for the analysis were sperm count after preparation (× 10^6^/ml), number of follicles > 16 mm at IUI, the time of hCG injection, male-factor and unexplained-infertility diagnosis and cycle day of insemination. All the factors were treated as dichotomous variables, except the cycle day of insemination, which was a continuous variable. The categories for dichotomous variables were: sperm count < or ≥ 20 × 10^6^/ml; number of follicles 0-1 or 2-3; the time of hCG injection either 24-32 h prior to IUI or just after IUI; male-factor (either male or non-male) and unexplained infertility (either unexplained or non-unexplained). The selected cut-off points for sperm count and number of follicles were arbitrarily chosen. GEE was performed using the SPSS 16.0 for Mac (SPSS, Chicago, IL). The chosen level of significance was P < 0.05.

## Results

The statistical analysis consisted of 332 intrauterine inseminations. There were 99 patients who had two inseminations and 134 who had only one insemination. The urinary pregnancy test yielded a positive result in 45 cycles, giving a pregnancy rate of 13.6%. The clinical pregnancy rate was 12.3%. In 25 out of 332 cycles (7.5%) all the follicles were already ruptured at the time of insemination; the urinary pregnancy test became positive in four cycles.

There were differences between those who conceived and who did not (Table 1.). Those who conceived were more likely to have unexplained infertility and less likely to have male factor as the main diagnosis. A sperm count ≥ 20 × 10^6^/ml after preparation and a tendency to have 2-3 follicles at IUI were more typical in women who became pregnant. In addition, hCG was injected more frequently after IUI in patients with a positive pregnancy test (Table 1.).

**Table 1:** Comparison of characteristics between those who tested positive and negative in urinary pregnancy test.

	Preg. test + (n = 45)	Preg. test - (n = 287)	*P*
Age (SD) in years	29.8 (5.1)	30.1 (4.3)	NS
Main diagnosis			
Unexplained	65.9%	47.3%	0.024
Male factor	6.8%	18.8%	0.053
Endometriosis	4.5%	8.7%	NS
Hormonal	13.6%	17.7%	NS
Multiple	4.5%	6.5%	NS
Sperm count ≥ 20 × 10^6^/ml after preparation	80.0%	61.1%	0.019
Total FSH consumption (SD)	155 IU (58)	160 IU (91)	NS
2-3 follicles at insemination	64.4%	49.1%	0.076
Cycle day of insemination (SD)	13.7 (1.7)	13.8 (1.6)	NS
hCG injection after IUI	46.7%	28.9%	0.024

### HCG administration before vs. after IUI

There were 228 inseminations where the hCG was injected 24-32 h before the IUI and 104 cycles where the injection was done just after the IUI. The comparison between the groups did not reveal any differences in female age, main infertility diagnosis, sperm count after preparation or number of follicles at the time of insemination (Table 2.). The pregnancy rate was 10.9% when hCG was injected before and 19.6% when hCG was injected after the IUI (*P *= 0.040). The clinical pregnancy rates were 9.6% and 18.3% (*P *= 0.032), respectively.

**Table 2:** Comparison of characteristics in patients with different timing in hCG injection

	hCG injection 24-32 h before IUI (n = 228)	hCG injection after IUI (n = 104)	*P*
Age (SD) in years	30.3 (4.4)	29.5 (4.5)	NS
Main diagnosis			
Unexplained	50.2%	50.0%	NS
Male factor	17.6%	16.7%	NS
Endometriosis	6.8%	10.2%	NS
Hormonal	16.7%	17.6%	NS
Multiple	7.2%	3.7%	NS
Sperm count ≥ 20 × 10^6^/ml after preparation (SD)	65.5%	62.2%	NS
Total FSH consumption (SD)	163 IU (98)	152 IU (58)	NS
Number of follicles >16 mm at insemination (SD)	1.5 (0.8)	1.6 (0.7)	NS
2-3 follicles at insemination	50.0%	53.2%	NS
Cycle day of insemination (SD)	13.9 (1.7)	13.5 (1.3)	0.014
Positive urinary pregnancy test	10.9%	19.6%	0.040
Clinical pregnancy rate	9.6%	18.3%	0.032

### Generalized estimating equations (GEE)

The aim of the GEE analysis here was to find independent factors affecting the cycle outcome. Data for 322 cycles were available and included in the final model (data of 10/332 cycles were incomplete). When the response was positive pregnancy test, the independent factors in the final model were sperm count, number of follicles and the time of hCG injection (Table 3). Male factor, unexplained infertility or the insemination cycle day were not independent factors and were, therefore, not included in the model. When the response was set as clinical pregnancy, the independent factors were sperm count and the time of hCG injection (Table 4.).

**Table 3:** GEE (n = 322) for positive pregnancy test: three independent factors significantly contribute to positive pregnancy test.

*Variable*		*OR (95% CI)*	*P value*
Sperm count after preparation	≥ 20 × 10^6^/ml vs. < 20 × 10^6^/ml	2.65 (1.20-5.81)	0.015
Number of follicles at insemination	2-3 vs. 0-1	2.01 (1.07-3.81)	0.031
Time of hCG injection	after IUI vs. 24-32 h before IUI	2.21 (1.16-4.19)	0.016

OR, odds ratio; CI, confidence interval.

**Table 4:** GEE (n = 322) for clinical pregnancy revealed two independent factors, which significantly contributed to clinical pregancy after IUI cycle.

*Variable*		*OR (95% CI)*	*P value*
Sperm count after preparation	≥ 20 × 10^6^/ml vs. < 20 × 10^6^/ml	2.21 (1.02-4.82)	0.045
Number of follicles at insemination	2-3 vs. 0-1	1.88 (0.98-3.61)	0.057
Time of hCG injection	after IUI vs. 24-32 h before IUI	2.11 (1.11-4.05)	0.025

OR, odds ratio; CI, confidence interval

## Discussion

Postponing the hCG administration until after the IUI instead of injecting it 24-32 hours before IUI resulted in a significantly increased pregnancy rate. The other independent factors affecting the IUI cycle outcome in this study were the number of follicles > 16 mm and the sperm count.

In most studies that have evaluated the outcome in IUI cycles, the insemination has been performed 24-36 hours following hCG administration [1,4]. This practice is based on data indicating that, in natural cycles, the ovulation takes place 32 hours (range 24-56 hours) after the onset of the luteinizing hormone (LH) surge [5], whereas in stimulated cycles, it takes place approximately 36-38 hours after the hCG injection [6]. As the current assumption is that the oocytes are fertilisable for only 12-16 hours [7] and the spermatozoa survive only for a limited period of time in the female reproductive tract [8-10], it is rational to schedule the insemination to the time of expected ovulation, i.e., 24-36 hours after the administration of hCG.

In 1995, Wilcox et al. published a study of 221 healthy women who were planning to become pregnant [3]. After stopping birth-control methods, the women collected daily urine specimens and kept daily records of their sexual intercourses. Oestrogen and progesterone metabolites were measured from the urine samples to estimate the day of ovulation. The authors observed that conception occurred only when intercourse took place during a six-day period that ended on the estimated day of ovulation [3]. This finding suggests that the chances to conceive in the natural cycle diminish considerably after ovulation and that, preferably, the spermatozoa should be available in the reproductive tract before ovulation occurs. It also suggests that spermatozoa may survive for several days after intercourse, as women whose last intercourse took place 5-6 days before ovulation did conceive.

The results of the study by Wilcox et al. (1995) do not totally support the current practice in IUI that hCG should be administered before the insemination but rather that it should be injected after the insemination. In our study, we observed an increase of 80% in pregnancy rate by postponing the hCG injection after the IUI. The change in hCG administration ensured that the spermatozoa were already present in the reproductive duct before ovulation took place, similar to the situation in successful natural cycles [3]. Since sexual intercourse was allowed a couple of days before the insemination and also after it, spontaneous conception is possible although unlikely, since all of the couples had at least one year infertility even before the start of basic investigations.

Our results also confirmed earlier findings that the number of follicles > 16 mm [11] and sperm count [12] are independent factors affecting the IUI cycle outcome.

## Conclusion

Postponing the hCG administration until after the IUI seems to increase considerably the pregnancy rate in IUI cycles. Since our study was retrospective and only limited number of outcomes were presented, the finding needs to be confirmed in randomised controlled trials. If the finding is confirmed, it may eventually lead to avoidance of long-lasting, inconvenient and expensive ovarian hyperstimulation and IVF/ICSI in some couples undergoing infertility treatment.

## Competing interests

The authors declare that they have no competing interests.

## Authors' contributions

IYJ has participated in designing the study, acquisition, analysis and interpretation of data, drafting and revising the article. JST has participated in interpretation of data and revising the article. HM has participated in acquisition and interpretation of data and revising the article. All the authors read and approved the final manuscript.
